# Surgical Treatment of Articular Cartilage Defects in the Knee: Are We Winning?

**DOI:** 10.1155/2012/528423

**Published:** 2012-05-13

**Authors:** A. R. Memon, J. F. Quinlan

**Affiliations:** Department of Trauma and Orthopaedics, Waterford Regional Hospital, Waterford, Ireland

## Abstract

Articular cartilage (AC) injury is a common disorder. Numerous techniques have been employed to repair or regenerate the cartilage defects with varying degrees of success. Three commonly performed techniques include bone marrow stimulation, cartilage repair, and cartilage regeneration. This paper focuses on current level of evidence paying particular attention to cartilage regeneration techniques.

## 1. Introduction

AC injury is a common disorder of the knee. It affects people of all ages and sexes. With the ever-increasing population and the active lifestyle of the older generation, incidence of AC injuries is on the rise. In the USA more than 500,000 procedures are performed for cartilage-related injuries and majority represent repeat procedures suggesting an ineffectiveness of surgical treatment [1]. The incidence of cartilage defects has been reported to be as high as 65% in routine knee arthroscopies [2–5]; however, the relevance of these defects to symptomatology is not yet clear. Hunter reported the inability of articular cartilage to regenerate in 1743 [6]. Early chondral lesions are often not detected due to lack of nerve supply, and the absence of vascularity limits the repair potential.

AC integrity is important for various reasons. Firstly, chondral lesions may cause mechanical symptoms such as swelling and pain. Secondly the progression to osteoarthritis is accelerated as reported by Mankin and Davis [7, 8]. Sahlstorm has reported radiographic evidence of OA in 100% patients in Ahlbeck stage II and III lesions at 20 years [9]. And thirdly the complexity of its structure and functional properties such as minimizing friction and increasing contact surface area to decrease wear under load bearing makes it a difficult material to repair.

AC works not only to protect the underlying subchondral bone but also serves to minimize friction and maximize load-bearing articular surface. Thus treatment of AC loss aims to restore these properties. AC is composed of chondrocytes (5–10%), water (65–80%), collagen, large negatively charged hydrophilic proteoglycans (aggrecan, hyaluronan), and smaller glycoproteins such as fibronectin and cartilage oligomeric proteins [10]. Microscopically, from superficial to deep, four distinct zones of AC are described [11]. The *superficial zone *is composed mainly of elongated chondrocytes. The smaller diameter collagen fibers (mainly type II) run parallel to the articular surface in this layer. The *transitional zone *is composed of large-diameter collagen fibers. The *deep zone* has perpendicularly arranged collages fibers and high proteoglycan content, and finally there is the *calcified cartilage zone*. Type II collagen is the predominant form (95%) but types VI, IX, X, and XI are also found (mostly in the calcified layer). As the chondrocytes move closer to the superficial zone, they become flatter in a fibroblastic shape. Such an arrangement of cells along with the collagen network in the superficial zone gives hyaline cartilage its resistance to shear forces, whereas in the superficial zone protein lubricant secreted by the chondrocytes reduces the coefficient of friction.

AC injury may be chondral or osteochondral if involving the underlying bone. The insult to AC can be traumatic or degenerative. Various metabolic factors such as obesity, alcohol abuse, and diabetes as well as mechanical factors like instability, trauma, and joint misalignment are implicated in its etiology. [12]. Pure chondral injures are painless and repair poorly due to lack of vascularity. Osteochondral injures heal by fibrocartilage secondary to initial inflammatory response. Although mesenchymal cells produce type I and II collagen, repair is mostly fibrocartilagenous in nature. It lacks the orderly structural organization of the normal hyaline cartilage and results in early degradation and fragmentation [13].

Both nonoperative and operative techniques have been employed in treating AC defects. The primary aim of any treatment modality is to reduce pain and restore function. Nonoperative treatments include weight loss, muscle strengthening physiotherapy, viscosupplementation with hyaluronic preparations, steroid injections, and oral chondroitin sulphate [14–17]. Operative treatment is broadly classified in three categories, namely, *bone marrow stimulation (BMS)* techniques, *cartilage replacement *techniques, and *cartilage regeneration *techniques. This paper aims to review the current concepts in the management of articular cartilage defects in the knee joint with particular emphasis on cartilage regeneration techniques.

## 2. Bone Marrow Stimulation (BMS) Techniques

### 2.1. Drilling/Microfracture/Abrasion Techniques

BMS techniques aim to stimulate the migration of mesenchymal stem cells to subchondral bone. Microfracture technique was first described by Steadman [18]. The scientific basis of this technique is to recruit the mesenchymal stem cells from to the surface of bleeding bone. They secrete fibrocartilage which is mainly composed of type I collagen. Such a repair tissue may be able to fill in the defect, but lacks the normal histological or biomechanical properties of hyaline cartilage. Therefore, it has inferior stability to compressive and shear forces and tends to deteriorate with time [19]. Younger age has been associated with better outcome. In a series of 72 patients younger than 45, Steadman has reported an improved outcome in 80% patients at 7 years [19]. Kreutz et al. have also shown better radiological fill of the cartilage defects with higher Cincinnati knee scores and ICRS in patients younger than 40 [20]. Decline in clinical outcome after 18–36 months is also more pronounced in older age individuals [20]. Comparison of BMS technique with repair or regenerative techniques is discussed further in the next sections.

## 3. Cartilage Replacement Techniques

### 3.1. Chondrocyte Autograft Transfer and Mosaicplasty

Two techniques described for cartilage replacement are Chondrocyte autograft transfer (OAT) and Mosaicplasty. OAT has been in practice since 1990s [21]. Osteochondral plugs are harvested from non-weight-bearing surface of the ipsilateral joint and placed in the prepared cylindrical hole in the region of cartilage defect. The limiting factor of size in OAT led to development of mosaicplasty, which harvests multiple small osteochondral plugs. Due to multiple cone implantations, the gaps between the plugs produce an uneven joint surface [22]. Microfracturing the gaps and insertion of Osteogenic Protein (BMP-7) have been used with varying results [23].

Due to the technical difficulties and donor site morbidity of mosaicplasty, its use is infrequent. However, a trend has been observed in single osteochondral grafts in isolated cartilage defects [24]. In a series of over 900 osteochondral grafts of the knee over a period of 15 years, Hangody et al. have reported good to very good results in 92% patients with femoral defects, 87% in tibia and 74% in patellofemoral joints [25]. Other authors have also reported 84 to 88% good to very good results at 2–4-year followup [26, 27]. When compared to Autologous Chondrocyte Implantation (ACI), Bentley has shown good to excellent results in 88% of patients with ACI compared to 69% following mosaicplasty [28]. On the other hand, Horas et al. have shown better clinical and histological outcome with mosaicplasty [29].

Complications of the osteochondral grafting include donor site morbidity with a 2.3% risk of patellofemoral arthritis [25]. Unsatisfactory filling of the cartilage defect (especially with grafts >8 mm in diameter) and fibrocartilage hypertrophy of the donor site have also been described [27, 30].

## 4. Cartilage Regeneration Techniques

### 4.1. Autologous Chondrocyte Implantation (ACI)

The most widely practiced cartilage regeneration technique is autologous chondrocyte implantation (ACI), which was first described by Brittberg in 1994 [31]. Since then ACI has gained widespread popularity. ACI involves harvesting the normal cartilage from a non-weight-bearing aspect of the ipsilateral joint. The chondrocytes are then expanded in vitro and subsequently implanted in the chondral defect. The main purpose of ACI is to implant chondrocytes in cartilage defect so that a hyaline cartilage is produced which closely resembles the normal AC in structural organization and functional characteristics. ACI is ideally used in femoral condylar AC defects, as results from patellar and tibial lesions are inconsistent. ACI is preferred in younger patients with no concomitant ligamentous instability and meniscal tears. Additional procedures including meniscectomy or ligament stabilization may be necessary to achieve optimum outcome. A rigorous postoperative rehabilitation regime is also followed for maximum benefit. Contraindications to ACI include history of septic arthritis and inflammatory arthritis such as rheumatoid.

## 5. Surgical Technique

ACI is a two-staged procedure. An initial arthroscopy is performed to evaluate the lesion. 3 to 4 chondral biopsies of AC are taken from non-weight-bearing surfaces of the joint (intercondylar notch, peripheral edges of femoral condyles). The specimen is then transported to the laboratory where the chondrocytes are isolated with an enzymatic process. The chondrocytes are then cultured for 3 to 4 weeks until volume increases by 30-fold for implantation (12 million chondrocytes approx.). Usually at 6 weeks from the initial surgery a second stage operation is carried out. Depending upon the location of the lesion, a medial or lateral patellar arthrotomy is performed. The defect is debrided and fashioned. A periosteal flap is then harvested from proximal tibia (medial femoral condyle can also be used). The flap is then secured to the defect (with its cambium layer facing the bone) on all sides except superiorly. The cultured chondrocytes are then injected under the flap and finally the flap is then attached superiorly as well. Fibrin glue may be used to seal the edges of the flap.

Postoperative rehabilitation involves early range of motion with protective weight bearing in a knee brace. Full weight bearing is usually achieved at 8 weeks. Subsequently light resistance and balance training is started. After 3 to 4 months strength gaining exercises are started and most physicians allow their patients to resume light athletic activities at 6 months. Return to full impact contact sport is usually not recommended until 12–18 months after-procedure.

## 6. Is ACI Effective?

Success of cartilage regeneration is based on its clinical, radiological, and histological outcome. In a number of observational studies, good to excellent clinical results have been obtained at short-to-medium-term followup [31–33]. When comparing ACI to OATS, Horas showed similar outcomes with both procedures; however, the speed of recovery was slower with ACI [29]. Bentley showed good to excellent results in 89% patients following ACI compared to 69% following mosaicplasty [28]. Dozin, on the other hand, observed improvement in 88% patients after mosaicplasty versus only 68% in ACI group [34]. Saris compared chondrocyte implantation with microfracture technique and found better cartilage histomorphometry at 12 months after chondrocyte implantation; however, no difference was observed in clinical outcome (KIIS, Knee Injury and Osteoarthritis Outcome score) at 12–18 months [35]. In another randomized controlled trial (RCT), Knutsen found no difference in clinical and radiological outcome between ACI and microfracture technique [36]. Comparing classical ACI using a periosteal flap with newer generation type I/III collagen membranes, Gooding et al. observed no difference in functional outcome at two years [37]. A similar RCT comparing ACI with matrix-induced ACI by Bartlett et al. also found no significant difference between the two techniques [38].

The drawbacks of ACI procedure include graft hypertrophy, which may need debridement (36% incidence). It involves two surgical procedures and is expensive to culture the chondrocytes in vitro. Due to the technical challenges of ACI procedure as well as the need for bigger surgical exposure, the need for an easier and effective method of injecting the chondrocytes into the joint has been long felt [38, 39]. This has led to the evolution of classical ACI into 2nd- and 3rd-generation procedures. 2nd-generation ACI used cultured chondrocytes but replaced the periosteal flap with a resorbable collagen matrix. In the 3rd-generation technique chondrocytes are seeded onto a type I/III collagen membrane and are then suspended into the defect site with fibrin glue. Amongst various commercially available membranes, the most commonly performed procedure is called matrix-induced autologous chondrocyte implantation (MACI, Genzyme Biosurgery, Cambridge, MA, USA). MACI not only has the advantages of reduced operating time [40] and reduced tourniquet time, it has also shown similar clinical results as traditional ACI.

The principle of MACI technique is to culture the chondrocytes onto a biocompatible three-dimensional scaffold (purified and cell free porcine collagen), which then would be implanted into the cartilage defect [41]. MACI still requires harvesting of chondrocytes from the native joint. The overall procedure is same as 1st-generation ACI except that instead of a periosteal flap, a three-dimensional scaffold of type I/III collagen is used. The scaffold is preseeded with chondrocytes. Cells grown on cartilage scaffold are capable of synthesizing chondrocyte matrix components such as chondroitin sulphate and glycosaminoglycans as well as S-100 protein (a cytoplasmic marker of chondrocytes) [42, 43]. The scaffold-chondrocyte complex is then directly applied to the AC defect after debridement and adhered with fibrin glue. A suture is sometimes necessary in large uncontained defects to secure the fixation. As extended dissection is not needed for harvesting a periosteal flap, this procedure can be done via miniarthrotomy or arthroscopy. After application of fibrin glue, pressure is applied for several minutes to obtain maximum adherence. The advantage of this technique is the shorter duration and that it can be performed at the same time of other procedures such as ACL reconstruction, high tibial osteotomy, and bone grafting [40].

Postoperatively, the joint is immobilized in extension. At 10 days, a supervised rehabilitation program is started. Continuous passive motion (CPM) has been shown to stimulate glycosaminoglycans, type II collagen, and chondroitin sulphate synthesis [44]. A protected weight-bearing regime is usually followed for eight to twelve weeks. The patient is not allowed to return to full sporting activities for approximately 18 months. Early accelerated rehabilitation (eight weeks) has shown no negative effect compared to delayed rehabilitation (eleven weeks) at short-term followup (3 months) [45]. Recently evidence has shown better clinical outcome with accelerated rehabilitation including early weight bearing and range of movement exercises [46–48].

## 7. Is MACI Superior to Other Cartilage Regeneration Techniques?

MACI is a newer technique and data regarding its efficacy is at best scarce. Several case series have described its efficacy and good short-term results; however, the long-term followup is lacking. Behrens, Elbert, Ventura, and Schneider et al. have described significant improvement in International Knee Documentation Committee (IKDC) Tegner Activity Score and Lysholm and Gillquist scores [46, 49–51]. However, randomised trials by Bartlett, Zeifang, and Manfredini comparing the outcome following MACI versus standard ACI using periosteal flap failed to show superiority of the MACI technique [38, 52, 53]. Zeifang et al. noted no difference in IKDC, Tegner Activity Score, and Short Form-36 at 12 and 24 months whereas better efficacy was observed with ACI technique on Lysholm and Gillquist scoring [52]. Bartlett et al. also observed no difference in the outcome following the two procedures [38]. Graft hypertrophy has been reported at almost 25% in MACI; however, it has not been associated with worse clinical outcome [54]. New experimental studies have been carried out in sheep models to replace the articular chondrocytes with predifferentiated mesenchymal stem cells. Initial results have shown good histological repair with less degradation at 1 year compared to classical articular chondrocyte [55]. This, if proved successful in humans, may substitute the need for the primary procedure to harvest the chondrocytes.

## 8. Conclusion

In conclusion, the search for ideal cartilage repair technique continues. The newer generation repair techniques have shown some promise, but long-term outcome is still unknown. Genetic modulation of mesenchymal stem cells and chondrocytes with viral and nonviral vectors has also shown potential but needs further evaluation. Long-term data is required to prove the real benefit of these costly interventions. While MACI has shown good early results, its long-term efficacy is unknown. Microfracture and abrasion arthroplasty are cheap and easier to do but do not provide a durable repair. OAT and mosaicplasty are extremely technically demanding with variable outcomes. Therefore articular cartilage repair remains under intense investigation and an ideal cure is yet to be defined.

##  Conflict of Interests 

 The authors declare no conflict of interests.
